# Pharmacokinetic/pharmacodynamic analysis of ceftazidime/avibactam and fosfomycin combinations in an *in vitro* hollow fiber infection model against multidrug-resistant *Escherichia coli*

**DOI:** 10.1128/spectrum.03318-23

**Published:** 2023-12-08

**Authors:** Niklas Kroemer, Lisa F. Amann, Aneeq Farooq, Christoph Pfaffendorf, Miklas Martens, Jean-Winoc Decousser, Nicolas Grégoire, Patrice Nordmann, Sebastian G. Wicha

**Affiliations:** 1 Institute of Pharmacy, University of Hamburg, Hamburg, Germany; 2 Dynamic Team – EA 7380, Faculté de Santé, Université Paris-Est-Créteil Val-De-Marne, Créteil, France; 3 Inserm U1070, Poitiers, France; 4 UFR de Médecine Pharmacie, Université de Poitiers, Poitiers, France; 5 Laboratoire de Toxicologie-Pharmacologie, CHU de Poitiers, Poitiers, France; 6 Medical and Molecular Microbiology, University of Fribourg, Fribourg, Switzerland; Hartford Hospital, Hartford, Connecticut, USA

**Keywords:** PK/PD, drug interactions, ceftazidime/avibactam, fosfomycin, synergy, hollow fiber infection model, *Escherichia coli*

## Abstract

Rational combination therapy offers a valuable option to increase efficacy and prevent emergence of resistance. Therefore, this study provides a translational pharmacokinetic/pharmacodynamic analysis of the synergy of /avibactam and fosfomycin in a clinical *Escherichia coli* strain expressing extended spectrum beta-lactamase (CTX-M-15 and TEM-4) and carbapenemase (OXA-244) genes. Detailed static time-kill experiments primed dynamic hollow fiber studies mimicking mono- and combination therapies with doses of ceftazidime/avibactam ranging from 0.06/0.015 to 2/0.5 g every 8 h (q8h) and doses of fosfomycin ranging from 0.125 to 6 g q8h. The drug effects and interactions were quantitatively evaluated by pharmacokinetic/pharmacodynamic modeling using semi-mechanistic and subpopulation synergy. A pharmacokinetic/pharmacodynamic model describing the effects of ceftazidime, avibactam, and fosfomycin and their synergy was developed from the static time-kill experiments and hollow fiber studies. Simulations revealed that combined doses as low as 0.5-g q8h fosfomycin and 0.25-/0.06-g q8h ceftazidime/avibactam lead to suppression of the bacterial count. Conversely, in monotherapy, substantially higher doses by a factor of 12 for fosfomycin (6 g q8h) or by a factor of 6 for ceftazidime/avibactam (1.5/0.375 g q8h) were needed to achieve a comparable killing over 72 h. The combination of ceftazidime/avibactam and fosfomycin was therefore shown to be highly synergistic and suppressed the emergence of resistances. Clinical evaluations of potential dose reductions or the possibility to treat strains with high-level resistance with this combination are warranted.

**IMPORTANCE:**

Mechanistic understanding of pharmacodynamic interactions is key for the development of rational antibiotic combination therapies to increase efficacy and suppress the development of resistances. Potent tools to provide those insights into pharmacodynamic drug interactions are semi-mechanistic modeling and simulation techniques. This study uses those techniques to provide a detailed understanding with regard to the direction and strength of the synergy of ceftazidime-avibactam and ceftazidime-fosfomycin in a clinical *Escherichia coli* isolate expressing extended spectrum beta-lactamase (CTX-M-15 and TEM-4) and carbapenemase (OXA-244) genes. Enhanced killing effects in combination were identified as a driver of the synergy and were translated from static time-kill experiments into the dynamic hollow fiber infection model. These findings in combination with a suppression of the emergence of resistance in combination emphasize a potential clinical benefit with regard to increased efficacy or to allow for dose reductions with maintained effect sizes to avoid toxicity.

## INTRODUCTION

The emergence of carbapenemase- and extended spectrum beta-lactamase (ESBL)-producing *Escherichia coli* and Enterobacterales, in general, is a threat to global health (1, 2). Besides the development of new antibiotics, rational combination therapy using marketed antibiotics is an option to increase efficacy due to synergistic interactions or to suppress the development of resistance and prolong the lifecycle of new agents (2).

A frequently evaluated drug for this purpose is fosfomycin (FOF) that has shown its synergistic potential in combination with other cell wall-interfering agents (3 – 5). Also, ceftazidime/avibactam (CZA) has already shown synergy for treating various multidrug-resistant Enterobacterales and *Pseudomonas aeruginosa* strains (6 – 8). Additionally, the common indications of CZA and FOF such as complicated intra-abdominal infections or urinary tract infections and hospital-acquired pneumonia, including ventilator-associated pneumonia, offer their use in combination. However, an evaluation on the clinical relevance of their drug interaction is lacking, and it remains unclear if the synergy could be exploited for dose reductions.

Pharmacokinetic/pharmacodynamic (PKPD) modeling is a state-of-the-art technique to translate the results from preclinical studies into the clinical setting (9, 10). With regard to drug combinations, PKPD models provide a semi-mechanistic understanding and quantification of drug interactions.

Hence, in this study, detailed static time-kill experiments were performed to elucidate the drug interactions of ceftazidime, avibactam, and FOF and evaluated by semi-mechanistic PKPD modeling. The model was then used to transfer the drug interactions into conceptual dynamic hollow fiber infection model (HFIM) experiments mimicking clinically achievable pharmacokinetics. Simulations were subsequently used to evaluate the potential for dose reductions in a combination therapy.

## RESULTS

### Bacterial isolate and susceptibility

The MICs against ceftazidime, CZA, and FOF of the clinical *E. coli* used in this study were 16, 0.125, and 16/0.5 mg/L (microdilution/agar dilution), respectively, and, therefore, classified as susceptible to CZA and FOF and resistant to ceftazidime alone according to the European Committee on Antimicrobial Susceptibility Testing (EUCAST) (11). Sequencing identified genes coding for CTX-M-15, TEM-4, and OXA-244 (OXA-48-like).

### Static time-kill experiments

**Fig 1:**
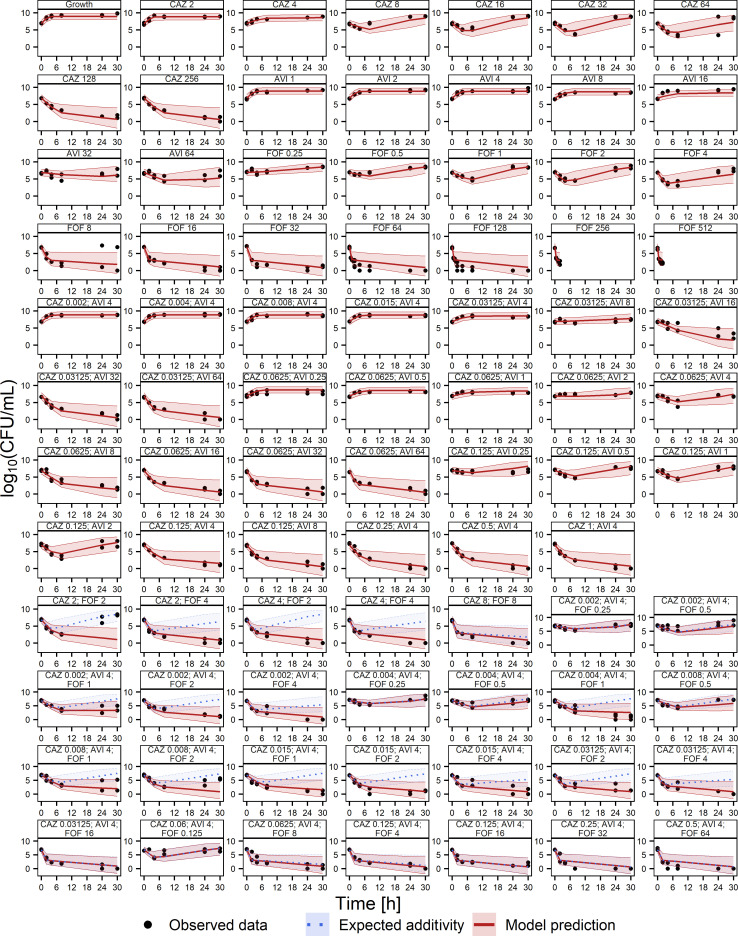
Stratified VPC (*n* = 1,000) on the static pharmacodynamic time-kill experiment model describing the bacterial count in log_10_ (CFU/mL) of the clinical *E. coli* isolate against ceftazidime (CAZ), avibactam (AVI), and FOF. Respective concentrations of the scenarios are given in milligrams per liter. Dots, observations; solid line, median prediction; dotted line, expected bliss independence; shaded areas, 90% prediction intervals.

Strong synergistic drug interactions were observed in the static time-kill experiments leading to enhanced killing effects in combination. In detail, concentrations of 128-mg/L ceftazidime or 16-mg/L FOF alone were required to achieve reproducible suppression of bacterial growth over 30 h, and no evaluated concentration of avibactam alone led to killing effects (Fig. 1). In contrast, combinations of 0.125-/4-mg/L CZA were efficacious. In combination with 2-mg/L FOF, even lower concentrations of CZA (0.002/4 mg/L) were sufficient to suppress the bacterial growth over 30 h.

### Dynamic HFIM

**Fig 2:**
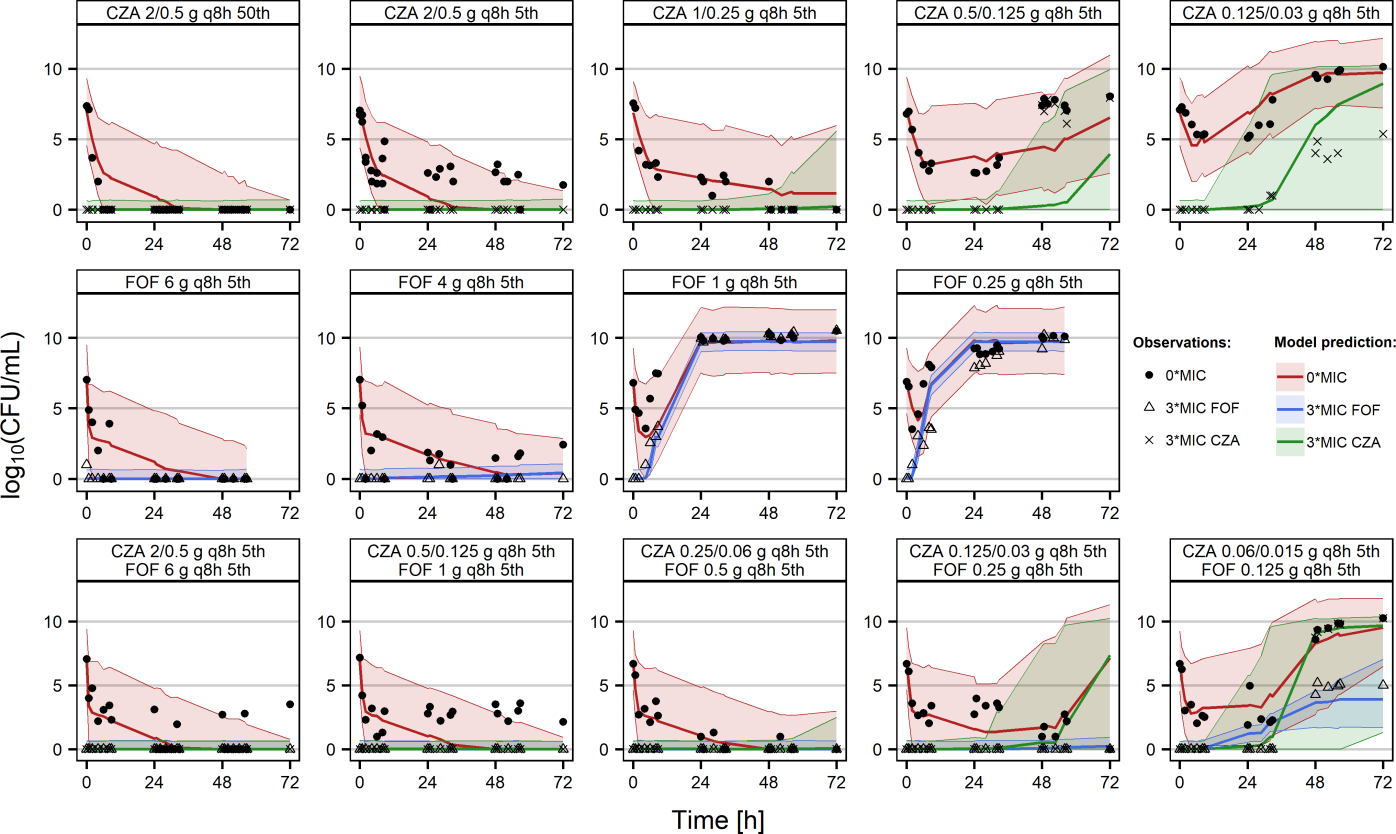
Stratified VPC (*n* = 1,000) on the PKPD model describing the bacterial count in log_10_ (CFU/mL) of the hollow fiber experiments of the clinical *E. coli* isolate against CZA and FOF (MIC_CZA_ = 0.125 mg/L; MIC_FOF_ = 16 mg/L). The percentiles (50th or 5th) of the doses correspond to the distribution of pharmacokinetic profiles, which would be expected from simulations of 1,000 patients given the defined dose. Dots, observations of different subpopulations; solid line, median prediction of different subpopulations; shaded areas, 90% prediction intervals of different subpopulations.

In the HFIM experiments, doses ranging from 6- to 0.125-g every 8 h (q8h) FOF and 2-/0.5- to 0.06-/0.015-g q8h CZA were mimicked. A dose of 1-g q8h FOF was the highest exposure tested not being able to reduce the bacterial count to or below the lower limit of quantification and prevent regrowth. Utilizing CZA in monotherapy, simulated doses of 0.5/0.125 g q8h displayed the highest exposure not suppressing regrowth. In combination, quarters of those doses (CZA 0.125/0.03 g q8h and FOF 0.25 g q8h) still achieved killing effects (Fig. 2). Below a certain exposure, a rapid emergence of 3× MIC FOF-resistant bacteria was observed within the first 12 h of experiment. On opposite, phenotypic resistance against 3× MIC CZA emerged later between 24 and 48 h.

The bioanalytical quantification of ceftazidime, avibactam, and FOF confirmed that the planned pharmacokinetic profiles in the HFIM experiment were adequately mimicked (Fig. S1). Therefore, the nominal pharmacokinetics were used for modeling and simulations of the HFIM data.

### PKPD modeling to quantify synergy and the tripartite effect relationship between CZA and FOF

**Fig 3:**
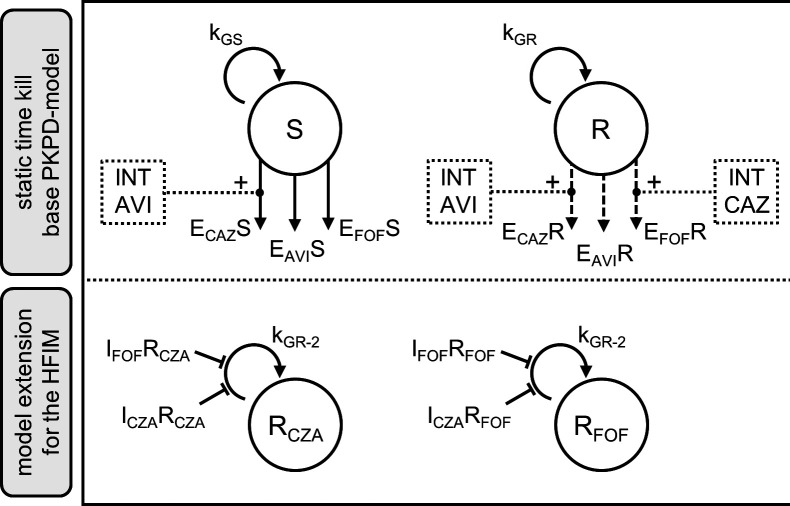
PKPD model structure combining elements of semi-mechanistic and subpopulation synergy. Upper part: model describing the static time-kill experiments. Lower part: model extension on the dynamic hollow fiber data. AVI, avibactam; CAZ, ceftazidime; CFU, sum of S + R (static time-kill curves) or sum of S + R + R_CZA_ + R_FOF_ (HFIM data); E_CAZ_R, E_AVI_R, E_FOF_R, effects on the resistant bacteria by the respective drugs; E_CAZ_S, E_AVI_S, E_FOF_S, effects on the susceptible bacteria by the respective drugs; I_CZA_R_FOF_, I_FOF_R_FOF_, growth inhibition of the phenotypic FOF less susceptible bacteria by the respective drugs; I_FOF_R_CZA_, I_CZA_R_CZA_, growth inhibition of the phenotypic CZA less susceptible bacteria by the respective drugs; INT, interaction; k_GR_, growth rate of the resistant bacteria; k_GR-2_, growth rate of the phenotypic-resistant bacteria; k_GS_, growth rate of the susceptible bacteria; R_CZA_, R_FOF_, phenotypic-resistant bacteria against the respective drugs. Dotted line, interaction direction; bold arrow, drug effects on the susceptible bacteria; dashed arrow, drug effects on the resistant bacteria; inhibition arrow, growth inhibition.

The effects of ceftazidime, avibactam, and FOF in the static time-kill curves were well described by a two-compartment model with a susceptible and a joint resistant subpopulation against CZA and FOF (Fig. 3). The individual bacterial killing effects against both subpopulations were mainly implemented as sigmoidal maximum effect models. Power models were only used to describe the drug effects of FOF on the susceptible subpopulation and of avibactam on the resistant subpopulation (Text S5). The general pharmacodynamic interaction (GPDI) model was able to capture synergies beyond bliss independence (12). In detail, those drug interactions were described best by a reduction of the EC_50_ on the susceptible and resistant populations of ceftazidime by avibactam by >99% and a reduction of the EC_50_ on the resistant population of FOF by ceftazidime by >99%. Interexperimental variability was implemented as exponential interindividual variability on the inoculum of the resistant population and on its growth rate. Visual predictive checks (VPCs) confirmed a high predictive performance of the static time-kill experiments (Fig. 1) but revealed a lack of predictability of the dynamic HFIM data, especially with regard to the rapid regrowth in the early phase of the experiments (0*–*12 h) or later when less susceptible subpopulations emerged and drove the regrowth profile (>30 h) (Fig. S2). Therefore, the model was further developed to fit the HFIM data by the addition of compartments describing the 3× MIC-resistant subpopulations and the suppression of those resistances in combination via subpopulation synergy (Fig. 2 and 3). Interexperimental differences in the regrowth behavior were captured by exponential interindividual variability on the inoculum of the resistant population and on the inoculum of the CZA less susceptible subpopulation. Details on the results of the modeling and the full-model parameters can be obtained from Text S5 and Tables S3 and S4.

### PKPD simulations to translate the synergy between CZA and FOF in the clinical perspective

**Fig 4:**
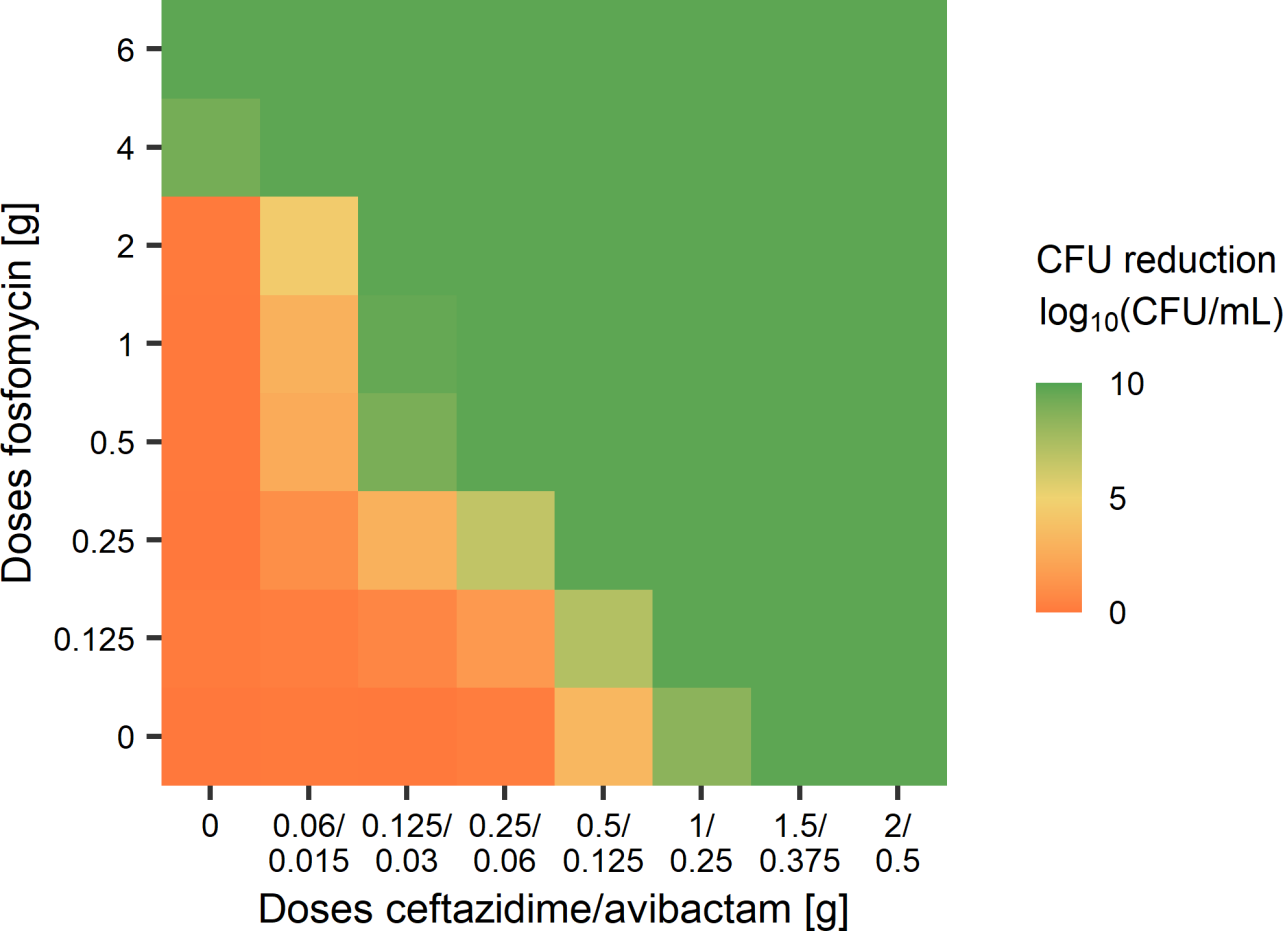
Dose-response surface of the outcome of hollow fiber experiments simulated with the dynamic PKPD model. Effect sizes are calculated as log_10_ (CFU/mL) reductions compared to no treatment after 72 h.

The dose-response surface of the simulations of additional HFIM experiments revealed the possibility for a dose reduction in combination to achieve the same outcome as the monotherapy (Fig. 4). In particular, a combination of 0.5-g q8h FOF and 0.25-/0.06-g q8h CZA would lead to a suppression of the bacterial count, while a 12 times higher dose of FOF (6 g q8h) or a six times higher dose of CZA (1.5/0.375 g q8h) would lead to the same effects in monotherapy.

## DISCUSSION

This PKPD study translated the strong synergy of CZA and FOF in a representative multidrug-resistant clinical *E. coli* from static into dynamic pharmacokinetic conditions. In opposite to an endpoint-driven evaluation of drug interactions, this study follows a translational approach based on clinical pharmacokinetics and evaluates the synergy of CZA and FOF in wide dose ranges with outcomes from suppression to regrowth of bacteria in static and dynamic time-kill experiments (13). Concentration-dependent emergence of resistance was identified as a missing link for the translation from static time kill to the HFIM. The emergence of resistance in the HFIM as well as the regrowth of bacteria after an initial decay in the static time-kill experiments occurred within the first 2 days of the experiments. This emphasizes the importance of an efficacious treatment away from the start of therapy. However, it cannot be ruled out that also doses suppressing the regrowth for 72 h would have allowed a delayed regrowth as a total eradication of bacteria was not investigated. Nevertheless, the suppression of the emergence of resistance in the static time-kill experiments as well as in the HFIM played a major role in the increased efficacy of the antibiotic drug combination and could potentially be explained by collateral sensitivity mechanisms and increased uptake of antibiotics when cell wall-interfering drugs are combined or by suppressed mutation frequencies (14 – 16). Commonly, higher mutation frequencies against FOF than against CZA are reported in Enterobacterales (17, 18). This nature was already confirmed for the evaluated *E. coli* in this study (*E. coli* YAL_AMA in Kroemer et al. (8)). Additionally, reduced mutation frequencies were identified against the combination of CZA and FOF. This is in line with the observed earlier resistance development against FOF in the hollow fiber experiments and, therefore, the significantly higher estimate of the inoculum of the phenotypically less susceptible subpopulation compared to CZA. The lower resistance development against CZA was additionally subjected to a higher variability. Therefore, interexperimental variability was needed in the model to describe the data. This led to relatively wide prediction intervals in the VPC plots covering the observed less susceptible bacteria against CZA. When implementing avibactam in PKPD models with ceftazidime, three major modes of action are conceivable: (i) own killing effect, (ii) potentiation of the ceftazidime effect, and (iii) suppression of beta-lactamase-mediated ceftazidime degradation. PKPD models have been developed including those three mechanisms (19, 20). In the present study, the bioanalysis identified no degradation of ceftazidime in the presence of the avibactam concentrations studied. Therefore, a permanent inhibition of the beta-lactamases by avibactam was assumed in this study, and a concentration-dependent inhibition was not implemented in the model. To reduce the complexity of the model, the avibactam effects were also simplified when modeling the suppression of the less susceptible subpopulations, and the combined suppressive effect of CZA was only described as a function of the ceftazidime concentrations. The results of the hollow fiber study and the simulations with the PKPD model emphasize enhanced killing effects leading to maintained high drug effects in combination at subinhibitory monotherapy exposures. This offers different potential benefits of a rational combination therapy. A rational combination therapy exploiting the synergistic effects could use lower doses while still being efficacious. Thereby, reduced drug exposures would avoid concentration-driven adverse effects such as neurotoxicity mediated by ceftazidime or hypokalemia mediated by FOF. In the presented study, the enhanced killing effects also prevented regrowth and the emergence of resistance. Thereby, also the combination of CZA and FOF against susceptible bacteria can be used to prolong the shelf life of CZA before resistances occur. Additionally, combination therapy could ensure maintained target site efficacy in critically ill patients with reduced drug exposures or altered target site pharmacokinetics. Lastly, synergistic drug effects could also contribute in re-sensitizing *E. coli* strains that are already resistant against monotherapy. In this context, it is important to note that the results of the HFIM study agree with the breakpoints for resistance set by EUCAST of >8 mg/L for CZA and >32 mg/L for FOF IV (11). The investigated *E. coli* strain is deemed susceptible to FOF and CZA according to EUCAST, and standard-dose monotherapies killed the bacteria and suppressed regrowth successfully.

We acknowledge the following limitations of our study: the dosing schemes were simplified for the HFIM experiments. The application of bolus injections in deviation to the clinical practice of 2-h infusions for CZA or 0.5-h infusions of FOF was driven by practicability reasons. Additionally, the focus on the reproduction of the peak (*C*_max_) and trough (*C*_min_) concentrations of the pharmacokinetic profiles enabled a simplified control of the elimination of all three drugs with a then-joint elimination half-life of approximately 2 h. This matches the clinically observed half-life for ceftazidime and avibactam, but the clinical half-life of FOF was shortened by 50% (21, 22). It could be expected to even increase the efficacy of the drug combination further, when also the dosing regimens have become a subject of optimization. So far, this study adds a conceptual approach to translating this drug interaction from static time-kill experiments into dynamic HFIM experiments.

This study was solely conducted as a single experiment in one clinical *E. coli* strain. However, previous research highlighted that the interactions of CZA and FOF behave relatively consistent among different clinical *E. coli* strains without an identified influence of the expressed genes (8). Hence, this study adds a conceptual demonstration of the synergy of CZA and FOF in an exemplary strain, but a confirmation of the synergy and further extrapolation to less susceptible or resistant strains would be desirable. The simulated pharmacokinetics were derived from published plasma pharmacokinetic models. Therefore, the target site exposition might be different, and additionally, host-bacteria interactions (e.g., the immune system) need to be considered when a clinical outcome is discussed. Nevertheless, clinical exploitation of the drug interaction might increase the robustness of the antibiotic therapy with regard to efficacy, prevention of the emergence of resistance, and tolerability against pharmacokinetic variability even in infections where a monotherapy could be sufficient.

To conclude, the presented translational *in vitro* study outlined the potential clinical benefit of the drug interaction of CZA and FOF in a clinical *E. coli* isolate. If the synergy demonstrated in *in vitro* experiments can be corroborated clinically, the combination of CZA and FOF comes with the potential for dose reductions or increased treatment success due to enhanced killing effects and suppression of the emergence of resistance. Perspectively, advanced dosing schemes as prolonged or continuous infusions should be evaluated to further benefit from the prevailing synergy of CZA and FOF. To be finally able to translate the drug interaction from “bench to bedside,” the development of a rational guidance like breakpoints of the drug combination or PKPD indices is required to advise clinical combination therapy.

## MATERIALS AND METHODS

### Strains, media, and antimicrobials

One clinical *E. coli* isolate carrying genes coding for both ESBL and carbapenemase was used. The strain was isolated from a rectal swab in a routine screening for multidrug-resistant bacteria in the Henri-Mondor Hospital in the East of Paris region. The genomes were assembled with shovill v1.0.4 (https://github.com/tseemann/shovill), and the resistome was identified using the ResFinder database available on the Center for Genomic Epidemiology platform (https://www.genomicepidemiology.org/).

Ceftazidime (Sigma-Aldrich, USA), avibactam (Sigma-Aldrich, USA), FOF (Sigma-Aldrich, USA), and glucose-6-phosphate (Sigma-Aldrich, USA) stock solutions were freshly prepared in sterile 0.9% NaCl solution and stored short term at −80°C.

The bacteria were cultivated on Columbia agar (Carl Roth, Germany). Serial dilution of bacterial samples and plating on Columbia agar plates containing no drug were used to determine total bacterial counts. Agar plates supplemented with 3× MIC were used for monitoring of emergence of phenotypically less susceptible subpopulations during the HFIM. Ceftazidime-containing agar plates were supplemented with a constant concentration of 4-mg/L avibactam. 25-mg/L glucose-6-phosphate was added to agar plates containing FOF corresponding to EUCAST recommendations.

The experiments were conducted in cation-adjusted Mueller-Hinton broth 2 (MHB) (Sigma-Aldrich, USA). In addition to the agar plates, MHB containing FOF was supplemented with glucose-6-phosphate. The final concentration of glucose-6-phosphate after all the dilution steps was kept at 25 mg/L.

### Susceptibility testing

Broth microdilution according to the CLSI guideline was applied for MIC determination (23). Turbidity endpoints were read for ceftazidime, CZA, and FOF after 24 h. Although microdilution is not the reference method for FOF susceptibility testing, threefold of the MIC determined by microdilution was used to monitor the emergence of phenotypic-resistant subpopulations of the *E. coli* strain in liquid growth medium during the hollow fiber experiments. To account for the elevated variability, the MIC determination was performed in triplicate, and the modal value was reported. Additionally, the MIC of FOF was determined by agar dilution.

### Static time-kill experiments

Static time-kill experiments over 30 h at 37°C ambient air were conducted to explore the pharmacodynamics of ceftazidime, avibactam, and FOF alone and in combination. The concentrations were selected covering full effect ranges from eradication to regrowth as well as clinically relevant concentrations. The time-kill experiments were performed with a total volume of 10 mL and were inoculated with 10^6^ CFU/mL. After 2 h of preincubation, the drugs were added. The bacterial counts were quantified at 0, 2, 4, 8, 24, and 30 h after addition of the drugs by serial dilution, plating, and manual colony counting. The lower limit of quantification for this method was around 10^1^–10^2^ CFU/mL. Not quantifiable bacterial counts were empirically set to 1 CFU/mL and included in the model-based data evaluation. The experiments were performed as duplicates.

### Dynamic HFIM

Dynamic HFIM experiments over 72 h at 37°C ambient air were utilized to investigate the pharmacodynamics of CZA and FOF and their drug interactions mimicking human pharmacokinetics (24). MHB was circulated from a central compartment through a hollow fiber cartridge (C2011; FiberCell Systems Inc., USA). Peristaltic pumps pumped fresh MHB from a reservoir into the central compartment, and the same volume was removed to control the pharmacokinetics of the drugs. The 40-mL extra-capillary space of the hollow fiber cartridge was inoculated with 10^6^ CFU/mL. After 2 h of preincubation, the first dose was administered by a syringe driver.

The total and phenotypic-resistant bacterial count against 3× MIC was quantified by sampling from the bacterial compartment, serial dilution, and plating of the dilutions on agar plates followed by manual counting. Alike in the static time-kill experiments, not quantifiable bacterial counts were empirically set to 1 CFU/mL and included in the model-based data evaluation. Samples to confirm the drug pharmacokinetics within the HFIM were drawn from the central compartment and stored at −80°C until analysis. Details on the HFIM experiments and the used equipment are described in Text S1.

### Pharmacokinetics

**Fig 5:**
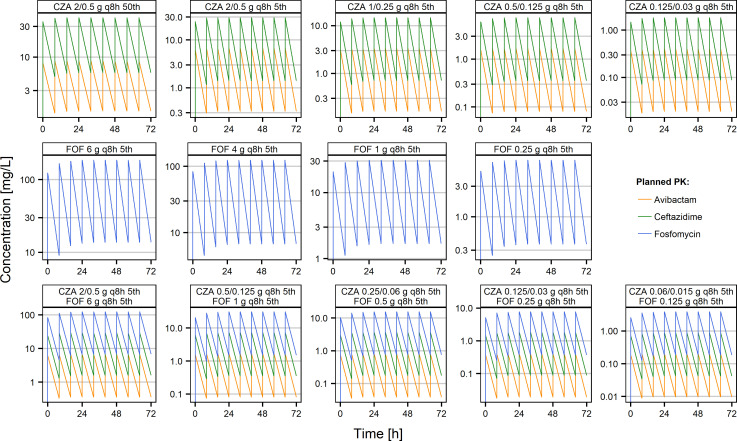
Overview of the simulated pharmacokinetics for the different hollow fiber experiments against CZA and FOF alone and in combination. The percentiles (50th or 5th) of the doses correspond to the distribution of pharmacokinetic profiles that would be expected from simulations of 1,000 patients given the defined dose. *C*_max_, maximum concentration at steady state; *C*_min_, minimum concentration at steady state.

Pharmacokinetic profiles of different CZA and FOF doses were simulated from published pharmacokinetic models derived from clinically observed drug concentrations (21, 22). From simulations of 1,000 virtual patients, defined percentiles were calculated to ensure that the mimicked pharmacokinetics profiles in the HFIM cover the clinically relevant concentration ranges. Simulated 50th percentiles thereby represent the antibiotic exposure within a typical patient, whereas the simulation of 5th percentiles represents the lower end of the exposure distribution in an inhomogeneous patient population, for example, due to altered pharmacokinetics by increased volume of distribution and/or clearance. In addition to standard doses, subtherapeutic doses were mimicked to evaluate the potential clinical relevance of the synergies. Dosing regimens with thrice daily doses q8h were focused, and for simplification, the drugs were administered to the HFIM as bolus injection maintaining the simulated peak (*C*_max_) and trough (*C*_min_) concentrations. The planned pharmacokinetics profiles are illustrated on Fig. 5, and the key pharmacokinetic properties are summarized in Table 1.

**TABLE 1:** Overview of the simulated pharmacokinetics for the different hollow fiber experiments against CZA and FOF alone and in combination^*a*^

Hollow fiber experiment	Avibactam (mg/L)		Ceftazidime (mg/L)		FOF (mg/L)		Simulated half-life (h)
	*C*_min_	*C*_max_	*C*_min_	*C*_max_	*C*_min_	*C*_max_	
CZA 2/0.5 g q8h 50th	1.43	8.71	5.70	41.11	-	-	3.03
CZA 2/0.5 g q8h 5th	0.32	6.69	1.40	29.06	-	-	1.81
CZA 1/0.25 g q8h 5th	0.16	3.34	0.71	14.66	-	-	1.81
CZA 0.5/0.125 g q8h 5th	0.08	1.67	0.35	7.33	-	-	1.81
CZA 0.125/0.03 g q8h 5th	0.02	0.40	0.09	1.83	-	-	1.81
FOF 6 g q8h 5th	-	-	-	-	13.67	185.37	2.10
FOF 4 g q8h 5th	-	-	-	-	6.74	124.31	1.88
FOF 1 g q8h 5th	-	-	-	-	1.68	31.08	1.88
FOF 0.25 g q8h 5th	-	-	-	-	0.37	7.72	1.81
CZA 2/0.5 g q8h 5th FOF 6 g q8h 5th	0.36	6.63	1.59	29.26	6.74	124.31	1.88
CZA 0.5/0.125 g q8h 5th FOF 1 g q8h 5th	0.08	1.67	0.35	7.33	1.50	31.12	1.81
CZA 0.25/0.06 g q8h 5th FOF 0.5 g q8h 5th	0.04	0.80	0.18	3.65	0.75	15.56	1.81
CZA 0.125/0.03 g q8h 5th FOF 0.25 g q8h 5th	0.02	0.40	0.09	1.83	0.37	7.72	1.81
CZA 0.06/0.015 g q8h 5th FOF 0.125 g q8h 5th	0.01	0.20	0.04	0.87	0.19	3.86	1.81

^*a*^ The percentiles (50th or 5th) of the doses correspond to the distribution of pharmacokinetic profiles that would be expected from simulations of 1,000 patients given the defined dose. *C*_max_, maximum concentration at steady state; *C*_min_, minimum concentration at steady state.

Protein binding for FOF is reported to be neglectable (25). For avibactam, a fraction unbound of 92% was assumed, and for ceftazidime, 85% fraction unbound was presumed as a compromise of the compared literature (21, 25).

### Bioanalysis

To confirm the pharmacokinetics of ceftazidime, avibactam, and FOF in the HFIM, samples covering the time course of the experiment were analyzed by ultra-high performance liquid chromatography-mass spectrometry (Text S2).

### PKPD modeling

In the first step, a semi-mechanistic PKPD model describing the static time-kill experiments was developed in NONMEM 7.5.0. (ICON, Gaithersburg, MD, USA) using second-order conditional estimation with interaction (LAPLACIAN-I). In brief, the monodrug effects of ceftazidime, avibactam, and FOF were modeled as sigmoidal maximum effect (*E*_max_) or power models on a two-compartment base model with susceptible and resistant subpopulations. Additivity was calculated by bliss independence (26, 27). Sequentially, drug interactions were introduced by the GPDI model (12). An increase of the ceftazidime potency mediated by avibactam and mono- and bidirectional drug interactions of ceftazidime and FOF was tested. Model selection was guided based on the Akaike information criterion, visual model fit, model stability, and condition number (28). To describe interexperimental variability, interindividual variability was tested on different growth parameters of the resistant population. The model was then evolved for the dynamic HFIM data. The pharmacodynamic parameters determined from the static experiments were fixed, and the model was extended to capture regrowth, which could not be mapped by the static time-kill PKPD model. Therefore, a submodel describing the emergence of phenotypic-resistant subpopulations against 3× MIC CZA or FOF was added. Drug effects were implemented as inhibition of the emergence of the resistances, and the drug interaction of ceftazidime and FOF was described by subpopulation synergy (29). Adjustments of the variability model with regard to interexperimental variability were again tested on growth parameters of the resistant population and the newly introduced subpopulations. Parameter uncertainty for both models was assessed by the sampling importance resampling routine implemented in Perl-speaks-NONMEM 5.0 (Uppsala University, Sweden) with the relative standard error calculated in the covariance step as proposal distribution (30). Details on the PKPD model building are described in Text S3.

### PKPD simulations

The dynamic PKPD model was then used for simulations of additional HFIM experiments. To evaluate the outcome of different dose combinations, the median reduction of the bacterial count after 72 h compared to no treatment was calculated.

## SUPPLEMENTAL MATERIAL

Supplemental material spectrum.03318-23-s0001.docx Text S1 to S5, Fig. S1 and S2, and Tables S1 to S4.

ASM does not own the copyrights to Supplemental Material that may be linked to, or accessed through, an article. The authors have granted ASM a non-exclusive, world-wide license to publish the Supplemental Material files. Please contact the corresponding author directly for reuse.
