# Supplementary material for: Pharmacokinetic/pharmacodynamic analysis of ceftazidime/avibactam and fosfomycin combinations in an in vitro hollow fiber infection model against multidrug-resistant Escherichia coli
Source: Microbiol Spectr. 2023 Dec 8;12(1):e03318-23. doi: 10.1128/spectrum.03318-23 (PMC10783110; doi:10.1128/spectrum.03318-23)
Supplement: Supplemental material — Text S1 to S5, Fig. S1 and S2, and Tables S1 to S4. [file spectrum.03318-23-s0001.docx]

***Microbiology Spectrum***Electronic Supplementary Material

**Pharmacokinetic/pharmacodynamic analysis of ceftazidime/avibactam and fosfomycin combinations in an *in vitro* hollow fiber infection model against multidrug-resistant *Escherichia coli***

Niklas Kroemer^a^, Lisa F. Amann^a^, Aneeq Farooq^a^, Christoph Pfaffendorf^a^, Miklas Martens^a^, Jean-Winoc Decousser^b^, Nicolas Grégoire^c,d,e^, Patrice Nordmann^f^, Sebastian G. Wicha^a,#^

^a^Dept. Of Clinical Pharmacy, Institute of Pharmacy, University of Hamburg, Germany

^b^Dynamic Team – EA 7380, Faculté de santé, Université Paris-Est-Créteil Val-De-Marne, France

^c^Inserm U1070, Poitiers, France

^d^Université de Poitiers, UFR de Médecine Pharmacie, Poitiers, France

^e^CHU de Poitiers, laboratoire de Toxicologie-Pharmacologie, Poitiers, France

^f^Medical and Molecular Microbiology, University of Fribourg, Fribourg, Switzerland

**^#^ Correspondance to:**

Prof. Dr. Sebastian G. Wicha

sebastian.wicha@uni-hamburg.de

Tel.: +49 40 42838-3487

Dept. of Clinical Pharmacy

Institute of Pharmacy

University of Hamburg

Bundesstr. 45

20146 Hamburg

Methods:

***Text S1: Methods of the hollow fiber infection model (HFIM)***

The HFIM system was conducted with polysulfone cartridges (C2011) purchased from FiberCell® Systems Inc. The central compartment had a volume of 200 mL and its circulation through the hollow fiber cartridge was ensured by a Duet Pump (FiberCell® Systems Inc., USA) with a flowrate of approximately 80 mL/min. The central compartment was connected with the hollow fiber cartridge by 4 m of oxygenator tubing. An Ismatec® Reglo ICC (12 rolls; VWR, USA) was used to pump fresh Mueller-Hinton-Broth 2 (MHB) (Sigma‑Aldrich, USA) from a reservoir into the central compartment with flow-rates ranging from 0.834 mL/min to 1.28 mL/min. The same volume was removed by a Masterflex L/S® digital pump drive (100 RPM, VWR, USA). Doses of the drugs were administered into the central compartment by a programmable syringe driver (Masterflex® Touch-Screen Syringe Pump 74905-54; VWR, USA). Because the elimination half-life of ceftazidime, avibactam and fosfomycin for the chosen conditions were approximately similar the HFIM setup was not augmented by additional dosing compartments (1). Before inoculation of the experiments the sterility of the cartridge and central compartment was checked by plating and incubation of samples of the respective compartments. The retention of the previously identified beta-lactamases CTX-M-15 and OXA-244 in the hollow fiber cartridge was corroborated exemplary by antigen tests of the bacterial suspension and the central compartment after 2 h of preincubation before the addition of the first dose. To identify the respective beta-lactamases the NG-Test ® CTX-M and NG-Test ® CARBA‑5 (NG Biotech, France) were used. Those tests identify CTX-M and OXA-48 like enzymes.

Up to 20 pharmacokinetic and pharmacodynamic samples were drawn at predetermined timepoints.
Pharmacodynamic samples were serially diluted and plated on drug-free agar plates and plates containing threefold minimum inhibitory concentrations (MIC). The growing colonies were counted after 24 h and 48 h respectively. The HFIM were performed as single experiments.

***Text S2: Methods of the bioanalysis***

The bioanalysis of the pharmacokinetic samples from the HFIM was performed to confirm the nominal concentrations and experimental conditions. To confirm the planned pharmacokinetic profiles, the drug concentrations at the beginning of each dosing interval (0.75 h time after dose) and mid-interval concentrations (4 h time after dose) were measured. The samples from the central compartment were stored at -80 °C after collection and thawed immediately before preparation. Calibration curves including seven calibration standards were prepared based on the expected drug concentrations in the HFIM experiments. The calibration curves ranged from 0.1 µg/mL to 40 µg/mL for ceftazidime, from 0.02 µg/mL to 10 µg/mL for avibactam and from 0.5 µg/mL to 50 µg/mL for fosfomycin. Samples expecting a higher fosfomycin concentration than 50 µg/mL were diluted 1:3 with MHB prior to sample preparation. To precipitate proteins 100 µL of sample were mixed with 50 µL internal standard solution (0.2 µg/mL moxifloxacin in methanol) and 300 µL ice-cold acetonitrile. The samples were then centrifuged at 17968 g at 4 °C for 20 min and 2 µL supernatant were directly injected on an Agilent 1290 Infinity 2 (Agilent Technologies, USA) UHPLC system coupled with a QTRAP 5500 (Sciex, USA) electrospray ionization mass spectrometer. Separation was achieved on a reverse phase Nucleodur PFP column (100x2 mm, 3 µm particle size; Macherey-Nagel, Germany) at 35 °C with a gradient elution. Solvents used were water containing 0.1% formic acid (A) and acetonitrile containing 0.1% formic acid (B). The gradient elution was performed as follows: starting conditions were 100% (A). After 0.8 min (B) linearly increased to 20% until 2 min and from there further increased to 40% (B) until 3.5 min, followed by isocratic elution with 40% (B) until 4.5 min and a return to starting conditions of 100% (A) after 4.6 min and reconditioning until 6 min. Fosfomycin and avibactam were detected within the first four minutes with the mass spectrometer operating in negative mode and then switched to positive mode for the detection of ceftazidime and moxifloxacin. Details on the ion source and detection parameters of the analytes are provided in Tables S1 and S2.

**Table S1:** Ion source parameters of the two different ESI phases of the method

|  | Curtain Gas (psi) | Collision Gas | Ion Spray Voltage (volt) | Temperature (°C) | Nebulizer Gas (psi) | Heater Gas (psi) |
| --- | --- | --- | --- | --- | --- | --- |
| Phase 1 (0-4 min) | 40 | Medium | -3500 | 450 | 50 | 50 |
| Phase 2 (4-6 min) | 20 | Medium | 4000 | 450 | 50 | 50 |

**Table S2:** Fragments and parameters used for each antibiotic and the internal standard (IS)*.* Individual standard solutions of each analyte and internal standard were directly injected to optimize their mass spectrometry parameters.

| Antibiotic | Identifier | ESI mode | Retention time (min) | Q1 (Da) | Q3 (Da) | Dwell Time (msec) | Declustering Potential (volts) | Entrance Potential (volts) | Collision Energy (volts) | Collision Cell Exit Potential (volts) |
| --- | --- | --- | --- | --- | --- | --- | --- | --- | --- | --- |
| Fosfomycin | Quantification | - | 1.22 | 136.884 | 78.9 | 150 | -100 | -10 | -62 | -13 |
| Fosfomycin | Qualification | - | 1.22 | 136.884 | 81.1 | 150 | -100 | -10 | -32 | -13 |
| Avibactam | Quantification | - | 2.60 | 263.913 | 80 | 150 | -80 | -10 | -38 | -11 |
| Avibactam | Qualification | - | 2.60 | 263.913 | 95.8 | 150 | -80 | -10 | -20 | -9 |
| Ceftazidime | Quantification | + | 4.29 | 546.957 | 468 | 150 | 96 | 10 | 17 | 20 |
| Ceftazidime | Qualification | + | 4.29 | 546.957 | 167.2 | 150 | 96 | 10 | 33 | 18 |
| Moxifloxacin (IS) | Quantification | + | 5.20 | 402.118 | 384.2 | 150 | 96 | 10 | 33 | 22 |
| Moxifloxacin (IS) | Qualification | + | 5.20 | 402.118 | 96.1 | 150 | 96 | 10 | 79 | 12 |

***Text S3: Methods of the pharmacokinetic/pharmacodynamic (PKPD) modelling***

**Static time kill experiments**

The PKPD model describing the static time kill data was built sequentially: in a first step the mono drug effects of ceftazidime, avibactam and fosfomycin were estimated, in a second step the mono drug parameters were fixed and solely the drug interaction parameters were estimated and in a final estimation step all parameters were unfixed and estimated together. The CVODES differential equation solver implemented as ADVAN14 in NONMEM® 7.5.0. (ICON, Gaithersburg, MD, USA) was used for model development. The base model consisted of a two-compartment ordinary differential equation system describing a susceptible (S) and a resistant (R) population affected by different drug effects (E_S_ and E_R_) (Eqs. 1-2). The growth kinetics of the bacteria were described by individual inocula of the two compartments and individual growth constants (k_GS_ and k_GR_). The growth of the bacteria was limited to a global capacity limit (B_max_).

| $\frac{dS}{dt}=S \cdot k_{GS} \cdot\left( 1-\frac{S+R}{B_{max}} \right)-S \cdot E_{S}$ | (1) |
| --- | --- |
| $\frac{dR}{dt}=R \cdot k_{GR} \cdot\left( 1-\frac{S+R}{B_{max}} \right)-S \cdot E_{R}$ | (2) |

The individual drug effects were preferably described by sigmoidal maximum effects models or power functions (Eqs. 3-4).

| $E(C) =\frac{Emax\cdot C^{H}}{{EC_{50}}^{H}+C^{H}}$ | (3) |
| --- | --- |
| $E(C) =Slope\cdot C^{H}$ | (4) |

with Emax being the maximum kill rate of the respective agent (h^-1^), C the concentration of the drug (mg/L), H the sigmoidicity parameter, the EC_50_ the concentration (mg/L) at which the drug effect is half-maximum and Slope being the linear correlation factor of the concentration to the killing rate of an agent (L/(mg x h)). The model selection was made based on graphical model fit, model stability and Akaike information criterion (AIC).

For combined drug effects the applied additivity criterion was Bliss Independence. To calculate the probabilistic Bliss Independence term for three drugs the single drug effects were normalized to the maximum drug effect of the combined drugs and afterwards scaled back to the effect scale (Eqs. 5-6) (2, 3).

| $E_{max}=max(E_{max,A},E_{max,B},E_{max,C})$ | (5) |
| --- | --- |
| $E_{comb}=\left( \frac{E_{A}}{E_{max}}+\frac{E_{B}}{E_{max}}+\frac{E_{C}}{E_{max}}-\frac{E_{A}}{E_{max}} \cdot\frac{E_{B}}{E_{max}}-\frac{E_{A}}{E_{max}} \cdot\frac{E_{C}}{E_{max}}-\frac{E_{B}}{E_{max}} \cdot\frac{E_{C}}{E_{max}}+\frac{E_{A}}{E_{max}} \cdot\frac{E_{B}}{E_{max}} \cdot\frac{E_{C}}{E_{max}} \right) \cdot E_{max}$ | (6) |

In cases, where the drug effects of ceftazidime, avibactam or fosfomycin were described by a power function effect sizes below 50% of the maximum effect were assumed. In these cases the probabilistic correction terms in Bliss Independence become neglectable and criterion collapses to simple addition of the effect sizes (Eq. 7) (3).

| $E_{comb}={E_{A}+ E}_{B}{+ E}_{C}$ | (7) |
| --- | --- |

Semi-mechanistic modelling of the drug interactions was performed by application of the general pharmacodynamic interaction (GPDI) model (4). The GPDI model describes drug interactions by the insertion of a GPDI-term shifting pharmacodynamic parameters (Θ) like Emax or EC_50_ affected by a present concentration of a combination partner (Eq. 8). Depending on the affected parameter and the polarity of the maximum interaction shift (INT) synergistic or antagonistic drug interactions can be modelled. The magnitude of the shift is depending from the concentration of the perpetrator drug (C; mg/L), the sigmoidicity of the interaction (H_INT_) and the potency of the interaction (EC_50-INT_; mg/L).

| $\Theta_{GPDI}= \Theta\cdot\left( 1+\frac{INT\cdot C^{H_{INT}}}{{EC}_{50-INT}^{H_{INT}}+C^{H_{INT}}} \right)$ | (8) |
| --- | --- |

If the GPDI-term is implemented on one combination partner, interactions are monodirectional. They can become bidirectional when the GPDI-term is implemented on both interaction partners. In those cases, both drugs are perpetrator and victim of the interaction at the same time and also asymmetric drug interactions with simultaneous synergy and antagonism are possible. For the drug interaction of avibactam and ceftazidime the interactions were implemented as shifts on the EC_50_ of ceftazidime mediated by avibactam.

For the drug interaction of ceftazidime and fosfomycin four different implementations of the GPDI model were tested before unfixing all mono drug effects for the final simultaneous estimation of all parameters: I) a monodirectional interaction of ceftazidime affecting fosfomycin, II) a monodirectional interaction of fosfomycin affecting ceftazidime, III) a bidirectional interaction described by one shared maximum interaction shift and IV) a bidirectional interaction described by separate maximum interaction shifts.

Uncertainty of the parameters of the static time kill model was assessed by the sampling importance resampling (SIR) routine implemented in Perl-speakes-NONMEM (PsN) 5.0 (Uppsala University, Sweden) with the relative standard errors (RSE) calculated in the covariance step as proposal distribution (5).

**Dynamic hollow fiber infection model**

The model developed based on the static time kill curve data was further developed to include the HFIM data. All parameters related to the drug effects were fixed. The ODE system was extended by two compartments describing the phenotypic less susceptible subpopulations which emerged against ceftazidime/avibactam or fosfomycin (R_CZA_ and R_FOF_) (Eqs. 9-12):

| $\frac{dS}{dt}=S \cdot k_{GS} \cdot\left( 1-\frac{S+R+R_{CZA}+R_{FOF}}{B_{max}} \right)-S \cdot E_{S}$ | (9) |
| --- | --- |
| $\frac{dR}{dt}=R \cdot k_{GR} \cdot\left( 1-\frac{S+R+R_{CZA}+R_{FOF}}{B_{max}} \right)-S \cdot E_{R}$ | (10) |
| $\frac{dR_{CZA}}{dt}=R_{CZA}\cdot k_{GR_{CZA/FOF}} \cdot\left( 1-\frac{S+R+R_{CZA}+R_{FOF}}{B_{max}} \right)\cdot\left( 1-E_{{CZA}_{R_{CZA}}} \right)\cdot(1-E_{{FOF}_{R_{CZA}}})$ | (11) |
| $\frac{dR_{FOF}}{dt}=R_{FOF}\cdot k_{GR_{CZA/FOF}} \cdot\left( 1-\frac{S+R+R_{CZA}+R_{FOF}}{B_{max}} \right)\cdot\left( 1-E_{{FOF}_{R_{FOF}}} \right)\cdot(1-E_{{CZA}_{R_{FOF}}})$ | (12) |

with additional effects of ceftazidime and fosfomycin supressing the emerging subpopulations (E_CZA_ and E_FOF_).

For suppression of resistance development, the maximum effect was assumed to be a full inhibition of the growth of the respective subpopulation. Therefore, a simplification of the sigmoidal maximum effect model was applied (Eq. 13).

| $E(C) =\frac{C^{H}}{{EC_{50}}^{H}+C^{H}}$ | (13) |
| --- | --- |

The sigmoidicity parameters were either estimated or fixed to constants (i.e. 1 for model simplification or 20 for steep concentration effect relations). The inhibitory effects of the two drugs ceftazidime and avibactam were merged for the subpopulation synergy and the effect was estimated as a function of the concentration of ceftazidime.

To account for the lower limit of quantification of the less susceptible subpopulations a baseline was set to 0 log10(CFU/mL) and the time points of the emergence of resistance were solely driven by the inoculum of the respective subpopulation and the drug concentrations supressing their growth.

As described above the uncertainty of the parameters of the dynamic HFIM model was also assessed by the SIR routine implemented in PsN 5.0 (Uppsala University, Sweden) with the relative standard errors (RSE) calculated in the covariance step as proposal distribution (5).

Results:





**Figure S1:** Illustration of the quantified concentrations of ceftazidime (CAZ), avibactam (AVI) and fosfomycin (FOF). Planned pharmacokinetic (PK) profiles (lines) are compared to the measured concentrations (symbols). Facets A to E display the monotherapy simulations of ceftazidime/avibactam, facets F to I display monotherapy simulations of fosfomycin and facets J to N display combination therapy simulations. The percentiles represent the percentage of patients from 1000 simulations achieving the displayed or lower pharmacokinetic profiles.

***Text S4: Results of the dynamic hollow fiber infection model***

The antigen test against the beta-lactamases CTX-M-15 and OXA-244 after 2 h of preincubation confirmed the expression of the beta-lactamases and their retention in the hollow fiber cartridge.

The quantified drug concentrations matched the planned pharmacokinetic profiles and no extensive degradation of the ceftazidime by beta-lactamases was apparent.

***Text S5: Results of the pharmacokinetic/pharmacodynamic modelling***

**Static time kill experiments**

Comparable regrowth patterns of the bacteria incubated with the different drugs alone and in combination allowed for a combination of the emerging bacteria in one joint resistant (R) subpopulation. Most calculated drug effects of ceftazidime, avibactam and fosfomycin were supported by sigmoidal maximum effect models. Solely the drug effect of fosfomycin on (S) and the drug effect of avibactam on (R) were described by power models. Therefore, for the (S) population the Bliss Independence criterion collapsed to effect addition. Because of the small effect sizes of avibactam compared to ceftazidime and fosfomycin (Emax­­­_CAZ_: 0.659 h^-1^ and Emax­­­_FOF_: 0.635 h^-^1 compared to the avibactam effect at the highest applied concentration: 0.294 h^-1^), just the maximum effects of ceftazidime and fosfomycin were considered for the normalization for the calculation of Bliss Independence on the (R) population.

The exploratory graphical analysis of the drug interactions of avibactam with ceftazidime and ceftazidime with fosfomycin indicated maintained drug effects at significantly reduced drug concentrations (e.g. Fig. 1: ceftazidime 0.0625 µg/mL + avibactam 8 µg/mL had a similar effect as ceftazidime 128 µg/mL alone and ceftazidime 2 µg/mL + fosfomycin 4 µg/mL had a similar effect as fosfomycin 16 µg/mL alone). Therefore, the implementation of the drug interaction via the GPDI model was focused on EC_50_ interactions. A pharmacokinetic drug interaction of avibactam and ceftazidime by means of an inhibition of the degradation of ceftazidime by beta-lactamases was neglected, because of the absence of quantitative data for the static time kill experiments.

The drug interaction of ceftazidime and fosfomycin was described best by a mono directional interaction with a potentiation of the fosfomycin EC_50_ on the (R) population. The respective Akaike Information Criteria differences for the different GPDI model implementations computed against Bliss Independence were as follows:

Monodirectional interactions:
I) ceftazidime affecting fosfomycin: -271.991
II) fosfomycin affecting ceftazidime: -2.035

Bidirectional interactions:
III) interactions described by one shared maximum interaction shift: -262.994
IV) interactions described by separate maximum interaction shifts: -251.171

The model estimated strong synergistic interactions with maximum interaction shifts (INT) reducing the EC_50_ by > 99% for both interacting drug pairs (i.e. ceftazidime/avibactam and ceftazidime/fosfomycin). Those estimates are in line with the *in vitro* observed interaction effect sizes outlined above. To support the accurate estimation and to avoid boundary issues the INT-parameters and interaction potencies (EC_50_s of the interaction) were transformed to a logarithmic scale. This rescaling enabled the estimation of the full concentration-interaction-relations without any further assumptions. For example, the interaction of ceftazidime and avibactam is characterised by a very small EC_50_ of the interaction and a Hill factor of 0.266. This leads mathematically to an onset of the potentiation of ceftazidime by avibactam at very small concentrations followed by a less steep concentration-potentiation-relation when the avibactam concentration is further increased. This finding is in line with the *in vitro* observations. The full set of model parameters is displayed on Table S3.

Interexperiment variability was tested as interindividual variability on the inoculum and the growth rate of the (R) population and was implemented on both parameters as exponential coefficient of variation.

**Table S3:** Typical PD parameters (Θ) of the static PD model developed based on data of static time kill experiments including 95% confidence intervals obtained by the sampling importance resampling (SIR) technique.

| **Structural model parameters** | |
| --- | --- |
| Inoculum susceptible bacteria (S) [log_10_(CFU/mL)] | 6.86 [6.74-6.96] |
| Inoculum resistant bacteria (R) [log_10_(CFU/mL)] | 3.14 [2.81-3.43] |
| Maximum bacterial capacity [log_10_ (CFU/mL)] | 8.92 [8.76-9.06] |
| Growth rate (S) [h^-1^] | 1.81 [1.61-2.15] |
| Growth rate (R) [h^-1^] | 0.45 [0.41-0.52] |
| **Mono drug PD parameters** | |
| Emax of CAZ on (S) [h^-1^] | 3.40 [3.08-3.87] |
| EC_50_ of CAZ on (S) [mg/L] | 5.31 [4.23-6.54] |
| Hill factor of CAZ on (S) | 2.32 [1.77-2.88] |
| Emax of CAZ on (R) [h^-1^] | 0.659 [0.592-0.746] |
| EC_50_ of CAZ on (R) [mg/L] | 74.40 [64.25-92.04] |
| Hill factor of CAZ on (R) | 8.45 [5.98-10.95] |
| Slope of FOF on (S) [L/mg x h^-1^] | 2.71 [2.51-3.09] |
| Hill factor of FOF on (S) | 0.333 [0.292-0.377] |
| Emax of FOF on (R) [h^-1^] | 0.635 [0.582-0.718] |
| EC_50_ of FOF on (R) [mg/L] | 4.70 [3.67-5.72] |
| Hill factor of FOF on (R) | 4.08 [2.76-5.79] |
| Emax of AVI on (S) [h^-1^] | 3.3 [2.76-3.98] |
| EC_50_ of AVI on (S) [mg/L] | 22.3 [16.50-29.10] |
| Hill factor of AVI on (S) | 1.13 [0.92-1.39] |
| Slope of AVI on (R) [L/mg x h^-1^] | 0.0787 [0.0554-0.1101] |
| Hill factor of AVI on (R) | 0.317 [0.194-0.420] |
| **Interaction model: avibactam affecting ceftazidime** | |
| INT: maximum change of EC_50_ of CAZ on (S) mediated by AVI | -6.70 [-8.13- -5.65] ^1^ |
| EC_50_ of AVI in the interaction on EC_50_ of CAZ on (S) [mg/L] | -16.20 [-18.93- -13.99] ^2^ |
| Hill factor of AVI in the interaction on EC_50_ of CAZ on (S) | 0.266 [0.226-0.312] |
| INT: Maximum change of EC_50_ of CAZ on (R) mediated by AVI | -13.50 [-19.43- -9.69] ^1^ |
| EC_50_ of AVI of the interaction on EC_50_ of CAZ on (R) [mg/L] | -5.23 [-5.53- -5.04] ^2^ |
| Hill factor of AVI in the interaction on EC_50_ of CAZ on (R) | 1 ^3^ |
| **Interaction model: ceftazidime affecting fosfomycin** | |
| INT: Maximum change of EC_50_ of FOF on (R) mediated by CAZ | -7.85 [-10.08- -5.58] ^1^ |
| EC_50_ of CAZ in the interaction on EC_50_ of FOF on (R) [mg/L] | -12.40 [-15.01- -10.50] ^2^ |
| Hill factor of CAZ in the interaction on EC_50_ of FOF on (R) | 0.239 [0.179-0.311] |
| **Variability model** | |
| Inter-experimental variability on the inoculum of resistant bacteria (R) [%CV] ^4^ | 33.9 [27.8-38.9] |
| Inter-experimental variability on the Growth rate (R) [%CV] ^4^ | 22.6 [19.9-25.8] |
| Additive residual variability σ [log(CFU/mL)] | 1.30 [1.20-1.38] |

Abbreviations: AVI: avibactam; CAZ: ceftazidime; CFU: colony forming units; EC_50_: drug concentration at which the effect is half-maximum; Emax: maximum effect; FOF: fosfomycin; INT: maximum interaction shift

^1^ parameter was estimated on log scale TV = e^Θ^-1
^2^ parameter was estimated on log scale TV = e^Θ^
^3^ parameter was fixed to a constant
^4^ %CV was calculated as follows: $\%CV = \sqrt{\exp\left( \omega^{2} \right)-1} \cdot100\%$

**Dynamic hollow fiber infection model**

Simulations of the HFIM experiments using the static time kill PKPD model revealed, that the model solely informed by the static time kill experiments missed to capture rapid regrowth in the early phase of the HFIM experiments (0 h ‑ 12 h) or later regrowth profiles (> 30 h) (Figure S2). These regrowth patterns were driven by the emergence of phenotypic 3xMIC resistant subpopulations. Hence, the static time kill PKPD model was further developed to describe the dynamic HFIM experiments by the addition of two bacterial subpopulations describing the CFU growing on agar plates containing each drug at a concentration of 3xMIC. Those subpopulations were assumed to contribute to the total CFU count of the ODE system and allowed to quantify and accurately describe the emergence of 3xMIC resistance against the present antibiotics in the respective experiments.
To account for different growth conditions in the HFIM due to a constant supply of growth medium compared to static time kill experiments where the medium is not replenished, the bacterial inocula of the previous (S) and (R) populations as well as their growth rates and the maximum bacterial capacity were initially attempted to be estimated from the HFIM data. The data was insufficiently able to fully inform all growth parameters and the estimates tended towards the final static time kill PKPD parameter estimates. Therefore, both growth constants of the (S) and (R) populations as well as the inoculum of the (S) population were fixed, following previous studies where the phenomenon of non-diverging growth constants between static and dynamic experiments was also observed (6).
The model parameters are displayed on Table S4. The inoculum of the (R) population was estimated to be lower than for the static time kills curves (i.e. 2.89 log_10_(CFU/mL) instead of 3.14 log_10_(CFU/mL)), because the newly introduced phenotypic subpopulations covered parts of the regrowth pattern and the (R) population remained to describe lower resistance level besides the 3xMIC resistance. Describing the growth of the additional subpopulations, the additive residual error as well as the growth rates of both phenotypic less susceptible subpopulations were estimated to be similar. Therefore, they were merged to one respective parameter describing the residual error and growth rate of both phenotypic less susceptible subpopulations. Of note, the final estimate of the growth rate of the less susceptible subpopulations unexpectedly exceeds the growth rate of the (S) population (2.37 h^‑1^ against 1.81 h^-1^) (Tables S3, S4). Nevertheless, the confidence intervals of both estimates overlap, thus, a significant difference of the growth rates cannot be concluded. The relatively similar values indicate a low biological cost of the resistance development against ceftazidime/avibactam and fosfomycin.

The inoculum of the phenotypic resistant bacteria against ceftazidime/avibactam was fixed to the final estimate to stabilize the model. The very low inoculum of 10^-18^ CFU/mL corresponds to the later observed emergence of resistances against ceftazidime/avibactam compared to fosfomycin and allows to describe the higher variability of the emergence of resistance against ceftazidime/avibactam.

Regarding the effects suppressing the growth of the phenotypically resistant bacteria, the sigmoidicity parameters were either fixed to 1 or freely estimated. For very steep concentration effect relations the parameter was empirically fixed to 20.
Adjustments were made to the variability model to explain the observed interexperimental variability especially for the development of phenotypic resistances against ceftazidime/avibactam. The interindividual variability on the growth constant of (R) estimated for the static time kill data was not necessary to adequately describe the HFIM data. In contrast an interindividual variability on the inoculum of the phenotypic less susceptible subpopulation against ceftazidime/avibactam was implemented to capture the observed variability of the resistance development.

**Table S4:** Typical PD parameters (Θ) of the dynamic time kill model based on data of dynamic hollow fiber experiments evolved from the model developed for static time kill curve (TKC) experiments (Table S3) including 95% confidence intervals obtained by the sampling importance resampling (SIR) technique.

| **Structural model parameters** | |
| --- | --- |
| Inoculum susceptible bacteria (S) [log_10_(CFU/mL)] | 6.86 FIX to TKC parameter |
| Inoculum resistant bacteria (R) [log_10_(CFU/mL)] | 2.89 [2.68-2.99] |
| Maximum bacterial capacity [log_10_ (CFU/mL)] | 9.73 [9.50-9.91] |
| Growth rate (S) [h^-1^] | 1.81 FIX to TKC parameter |
| Growth rate (R) [h^-1^] | 0.45 FIX to TKC parameter |
| **Mono drug PD parameters** | |
| Emax of CAZ on (S) [h^-1^] | 3.40 FIX to TKC parameter |
| EC_50_ of CAZ on (S) [mg/L] | 5.31 FIX to TKC parameter |
| Hill factor of CAZ on (S) | 2.32 FIX to TKC parameter |
| Emax of CAZ on (R) [h^-1^] | 0.659 FIX to TKC parameter |
| EC_50_ of CAZ on (R) [mg/L] | 74.40 FIX to TKC parameter |
| Hill factor of CAZ on (R) | 8.45 FIX to TKC parameter |
| Slope of FOF on (S) [L/mg x h^-1^] | 2.71 FIX to TKC parameter |
| Hill factor of FOF on (S) | 0.333 FIX to TKC parameter |
| Emax of FOF on (R) [h^-1^] | 0.635 FIX to TKC parameter |
| EC_50_ of FOF on (R) [mg/L] | 4.70 FIX to TKC parameter |
| Hill factor of FOF on (R) | 4.08 FIX to TKC parameter |
| Emax of AVI on (S) [h^-1^] | 3.3 FIX to TKC parameter |
| EC_50_ of AVI on (S) [mg/L] | 22.3 FIX to TKC parameter |
| Hill factor of AVI on (S) | 1.13 FIX to TKC parameter |
| Slope of AVI on (R) [L/mg x h^-1^] | 0.0787 FIX to TKC parameter |
| Hill factor of AVI on (R) | 0.317 FIX to TKC parameter |
| **Interaction model: avibactam affecting ceftazidime** | |
| INT: maximum change of EC_50_ of CAZ on (S) mediated by AVI | -6.70 FIX to TKC parameter ^1^ |
| EC_50_ of AVI in the interaction on EC_50_ of CAZ on (S) [mg/L] | -16.20 FIX to TKC parameter ^2^ |
| Hill factor of AVI in the interaction on EC_50_ of CAZ on (S) | 0.266 FIX to TKC parameter |
| INT: maximum change of EC_50_ of CAZ on (R) mediated by AVI | -13.50 FIX to TKC parameter ^1^ |
| EC_50_ of AVI in the interaction on EC_50_ of CAZ on (R) [mg/L] | -5.23 FIX to TKC parameter ^2^ |
| Hill factor of AVI in the interaction on EC_50_ of CAZ on (R) | 1 FIX to TKC parameter |
| **Interaction model: ceftazidime affecting fosfomycin** | |
| INT: maximum change of EC_50_ of FOF on (R) mediated by CAZ | -7.85 FIX to TKC parameter ^1^ |
| EC_50_ of CAZ in the interaction on EC_50_ of FOF on (R) [mg/L] | -12.40 FIX to TKC parameter ^2^ |
| Hill factor of CAZ in the interaction on EC_50_ of FOF on (R) | 0.239 FIX to TKC parameter |
| **Less susceptible subpopulation model** | |
| Inoculum ceftazidime/avibactam less susceptible subpopulation [log_10_(CFU/mL)] | -18 ^3^ |
| Inoculum fosfomycin less susceptible subpopulation [log_10_(CFU/mL)] | -2.15 [-2.98- -1.52] |
| Growth rate less susceptible subpopulations [h^-1^] | 2.37 [2.09-2.68] |
| EC_50_ of FOF suppressing the FOF less susceptible subpopulation [mg/L] | 6.84 [6.48-7.17] |
| Hill factor of FOF suppressing the FOF less susceptible subpopulation | 20 ^4^ |
| EC_50_ of CZA suppressing the CZA less susceptible subpopulation [mg/L] | 0.576 [0.441-0.765] |
| Hill factor of CZA suppressing the CZA less susceptible subpopulation | 1 ^4^ |
| EC_50_ of CZA suppressing the FOF less susceptible subpopulation [mg/L] | 0.049 [0.040-0.057] |
| Hill factor of CZA suppressing the FOF less susceptible subpopulation | 2.49 [1.76-4.20] |
| EC_50_ of FOF suppressing the CZA less susceptible subpopulation [mg/L] | 1.38 [1.00-2.49] |
| Hill factor of FOF suppressing the CZA less susceptible subpopulation | 20 ^4^ |
| **Variability model** | |
| Inter-experimental variability on the inoculum of resistant bacteria (R) [%CV] ^5^ | 48.2 [40.4-56.9] |
| Inter-experimental variability on the on the inoculum of the ceftazidime/avibactam less susceptible subpopulation [%CV] ^5^ | 47.7 [28.6-65.9] |
| Additive residual variability on the total bacterial count σ [log(CFU/mL)] | 3.28 [2.98-3.67] |
| Additive residual variability on less susceptible σ [log(CFU/mL)] | 0.906 [0.837-1.00] |

Abbreviations: AVI: avibactam; CAZ: ceftazidime; CFU: colony forming units; CZA: ceftazidime/avibactam; EC_50_: drug concentration at which the effect is half-maximum; Emax: maximum effect; FOF: fosfomycin; INT: maximum interaction shift; TKC: static time kill curve

^1^ parameter was estimated on log scale TV = e^Θ^-1
^2^ parameter was estimated on log scale TV = e^Θ^
^3^ parameter was fixed to final estimate
^4^ parameter was fixed to a constant
^5^ %CV was calculated as follows: $\%CV = \sqrt{\exp\left( \omega^{2} \right)-1} \cdot100\%$


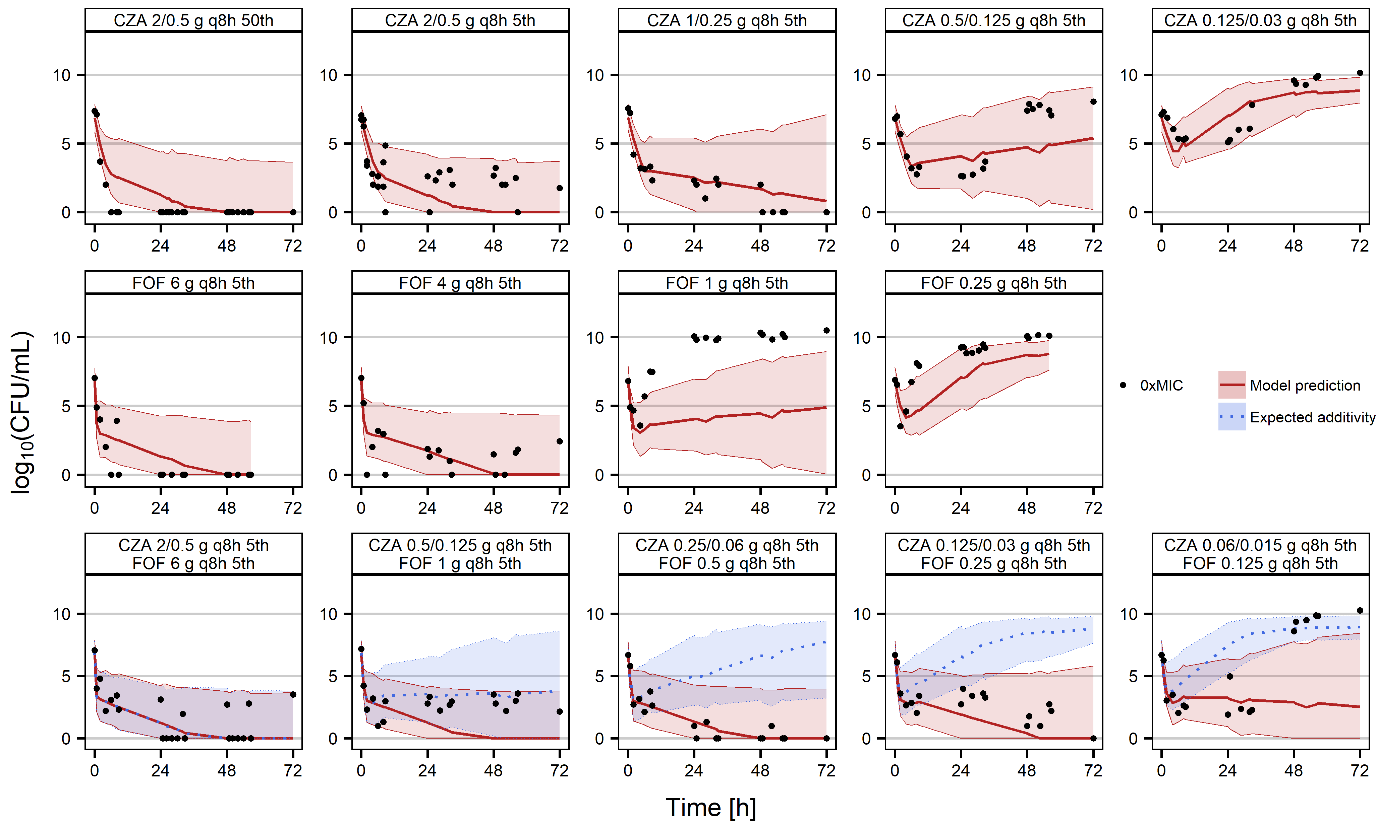


**Figure S2:** Stratified visual predictive check (VPC) (n=1000) on the PKPD model developed on static time kill experiments applied to the experimental data of the dynamic hollow fiber experiments. The percentiles (50^th^ or 5^th^) of the doses correspond to the distribution of pharmacokinetic profiles which would be expected from simulations of 1000 patients given the defined dose. Dots: observed bacterial count; solid line: median prediction; dotted line: expected Bliss Independence; shaded areas: 90% prediction intervals.

References

1. Sy SKB, Zhuang L, Sy S, Derendorf H. 2019. Clinical Pharmacokinetics and Pharmacodynamics of Ceftazidime–Avibactam Combination: A Model-Informed Strategy for its Clinical Development. Clin Pharmacokinet 58:545–564.

2. Goldoni M, Johansson C. 2007. A mathematical approach to study combined effects of toxicants in vitro: Evaluation of the Bliss independence criterion and the Loewe additivity model. Toxicol In Vitro 21:759–769.

3. Chen C, Wicha SG, De Knegt GJ, Ortega F, Alameda L, Sousa V, De Steenwinkel JEM, Simonsson USH. 2017. Assessing pharmacodynamic interactions in mice using the multistate tuberculosis pharmacometric and general pharmacodynamic interaction models. CPT Pharmacomet Syst Pharmacol 6:787–797.

4. Wicha SG, Chen C, Clewe O, Simonsson USH. 2017. A general pharmacodynamic interaction model identifies perpetrators and victims in drug interactions. Nat Commun 8:2129.

5. Dosne AG, Bergstrand M, Harling K, Karlsson MO. 2016. Improving the estimation of parameter uncertainty distributions in nonlinear mixed effects models using sampling importance resampling. J Pharmacokinet Pharmacodyn 43:583–596.

6. Nielsen EI, Cars O, Friberg LE. 2011. Predicting In Vitro Antibacterial Efficacy across Experimental Designs with a Semimechanistic Pharmacokinetic-Pharmacodynamic Model. Antimicrob Agents Chemother 55:1571–1579.
